# Effects of Ballroom Dance on Physical Fitness and Reaction Time in Experienced Middle-Aged Adults of Both Genders

**DOI:** 10.3390/ijerph18042036

**Published:** 2021-02-19

**Authors:** Valerio Bonavolontà, Francesca Greco, Umberto Sabatini, Francisco J. Saavedra, Francesco Fischetti, Carlo Baldari, Laura Guidetti, Maria Grazia Vaccaro, Gian Pietro Emerenziani

**Affiliations:** 1 Department of Basic Medical Sciences, Neurosciences and Sense Organs, School of Medicine, University of Bari “Aldo Moro”, 70124 Bari, Italy; francesco.fischetti@uniba.it; 2 Department of Experimental and Clinical Medicine, “Magna Graecia” University, 88100 Catanzaro, Italy; francescagreco1997@gmail.com (F.G.); emerenziani@unicz.it (G.P.E.); 3 Department of Medical and Surgical Sciences, Institute of Neuroradiology, Magna Græcia University, 88100 Catanzaro, Italy; sabatini@unicz.it; 4 Research Centre for Sports Sciences, Health and Human Development Sport Sciences Department, University of Trás-os-Montes e Alto Douro, 5000-801 Vila Real, Portugal; fjfsaave@utad.pt; 5 Department of Theoretical and Applied Sciences, eCampus University, 22060 Novedrate, Italy; carlo.baldari@uniecampus.it; 6 “Niccolò Cusano” University, 00166 Rome, Italy; laura.guidetti@unicusano.it; 7 Neuroscience Centre, Magna Graecia University, 88100 Catanzaro, Italy; mg.vaccaro@unicz.it

**Keywords:** cognitive functions, aging, partnered dances, fall prevention, physical activity

## Abstract

Ballroom dance practice might play a pivotal role for successful aging, but its effects could differ depending on dancers’ experience level. The aim of this study was to investigate the effects of six months of ballroom dance (three times/w) on physical fitness and reaction time (RT) in 24 middle-aged adults who are experienced dancers (age: 59.4 ± 11.6 years). Body composition, handgrip test (HG), standing long-jump test (SLJ), step test (ST), one-legged stance balance test (OLSB), and RT were assessed before (T_0_) and after six months (T_6_) of dance practice. RT was re-evaluated four months later (T_10_). RT was significantly (p < 0.05) lower at T_6_ (221.2 ± 20.3 ms) and T_10_ (212.0 ± 21.9 ms) than T_0_ (239.1 ± 40,7 ms); no significant differences were found between T_6_ and T_10_. No significant differences were observed for all the other parameters between T_0_ and T_6_: weight and muscle mass were significantly lower (p < 0.01) in females than in males, and percentage of fat mass was significantly higher (p < 0.01) in females than in males. HG was significantly higher in males than females (p < 0.01). Results suggest that in experienced middle-aged adults of both genders, ballroom dance may positively influence RT, and this result could be maintained for four months.

## 1. Introduction

Aging is a life-long process characterized by a progressive loss in cognitive function and physical fitness (PF) [1]. As the mean age of the population is increasing, there is a greater proportion of older adults at risk for developing non-communicable disorders (NCDs) such as cardiovascular, respiratory diseases, diabetes, and some types of cancers [2]. It is also known that aging is associated with a progressive reduction in brain volume, especially in the prefrontal and temporal cortices [3]. Resnick et al. [4] have found that individuals who remain medically and cognitively healthy show a slower rate of brain atrophy compared to non-demented older individuals. Recently, it has become clear that the aging brain could regain neuroplasticity, confirming that these changes are age-related, but not entirely unavoidable. These brain age-related changes might influence subjects’ reaction time that is closely associated with the risk of multiple falls in older adults [5].

Moreover, low PF, such as lower limb strength and balance, and cognitive impairments might increase the risk of falls [6]. Therefore, it is important to participate in regular physical activity (PA), which leads to positive outcomes on PF increasing individuals’ quality of life [7,8] and help to contrast cognitive decline and neurodegenerative diseases [9,10]. Although regular PA has been shown to have many health benefits in older adults, this population remains physically inactive [11]. In particular, to improve the strength of the lower limbs, various relatively fast and stability-challenging movements should be suitable, such as dance movements [12]. Dance could be an easy access PA practice with high levels of enjoyment that increase the exercise adherence and improve individuals’ PF [13,14]. Thus, dance practice requires a considerable cognitive, physical, and emotional engagement that could induce positive functional adaptations potentially promoting health-related benefits in inexperienced older dancers [15,16]. Indeed, six months of dance practice is additionally recommended as a successful measure to counteract unfavorable effects of aging on the brain in the elderly [3]. Waltz, Tango, Viennese Waltz, Slow Foxtrot, and Quickstep (standard dances) belong to the ballroom dances characterized by different movements alternating musical rhythms given by sudden accelerations with instant pauses. Each of them has its peculiar characteristic necessary to perform the correct technique, and all of them are danced in pairs [17]. Males and females perform different movements according to their role during dancing, and this could result in different effects on their PF.

In particular, ballroom dance practice leads to improvements in perceived PF and cognitive functioning in novice (<1 year of dance) and experienced (>2 years of dance) dancers [18]. However, Lakes et al. [18] assessed both PF and cognitive functions using a survey. Kattenstroth et al. [19] showed that expert dancers had better performance than sedentary subjects in terms of expertise-related domains such as posture, balance, and reaction times. In addition to this previous article, the same authors [20] demonstrated that regular dance practice promoted postural, sensorimotor, and cognitive performances without affecting cardio-respiratory functions in older dancers who have not been involved in any regular dancing activity for 5 years. However, Kattenstroth et al. [20] did not study the effects of partnered ballroom dance but a dance that could be performed alone without a partner (Agilando^TM^), and no data regarding body composition and muscle strength were assessed.

In inexperienced dancers, scientific evidence showed that dance practice could induce brain plasticity, at both structural and functional levels [21,22]. Given the positive effects of dance on PF and cognitive functions in novel dancers, it could be possible that different results could appear in experienced dancers [23]. Indeed, different volume dance practice (years of expertise) might differently influence PF and cognitive functions in older adults. Consequently, subjects might reach a plateau on PF and cognitive functions at different times. Therefore, the aim of this study was to investigate the effects of six months of ballroom dance (from November 2018 to May 2019 and then after summer season) on PF and reaction time in experienced middle-aged dancers of both genders.

## 2. Materials and Methods

### 2.1. Participants

Thirty-one experienced middle-aged adults were enrolled for the study. Twenty-four participants (age: 59.4 ± 11.6. years, 11 females and 13 males) were evaluated at T_6_ and 18 participants at T_10_. All participants were recruited from the Dance School “Free Dance” of Catanzaro. Written informed consent was obtained from the participants before study participation. For this single-arm trial study, only healthy experienced dancers were enrolled (dance average years = 11 years). Indeed, all participants were clinically evaluated before participation to exclude any contraindication to PA by a medical doctor (e.g., functional inabilities, cardiovascular diseases, or prosthesis). None of the participants were assuming any drugs that could interfere with the intervention effects, nor they did perform other types of physical exercise in addition to ballroom training.

### 2.2. Procedures

Participants carried out their dance protocol three days a week for six months. Each dance class lasted one hour and half and consisted of different choreographies, which include various rhythmic and simple movements typically of ballroom/standard dances (Waltz, Tango, Viennese Waltz, Slow Foxtrot, and Quickstep). All these dance styles were performed during each class session. Therefore, the rhythms of the music were different within the same dance class (File S1).

Each dance class was composed of 15 min of warm-up at low intensity (1.6–2.9 METs), followed by 60 min of dance practice and 15 min of cool-down. Dance training was performed at moderate intensity (subjects’ average heart rate during dance practice equal to 68% of their maximum heart rate calculated as 220 minus age) and was measured by subjects’ heart rate (HR) using a HR monitor (RS 400, Polar Electro™, Kempele, Finland). Before (T_0_) and after six months (T_6_) of intervention, anthropometric characteristics, physical fitness (PF), and reaction time (RT) were evaluated. Moreover, RT was re-evaluated four months after the end of dancing practice (summer season) (T_10_). During the summer season, subjects were allowed to practice unsupervised free dance without being involved in any organized class. Prior to the first testing session, all participants took part in a rehearsal session to familiarize themselves with the PF tests. To increase the reliability of measurements, all subjects were tested at T_0_, T_6_, and T_10_ in the evening from 5.00 pm to 8.00 pm by the same qualified sport scientists; fasting time was two hours before the measurements. This study was conducted according to the guidelines of the Declaration of Helsinki and approved by the Regional Ethics Committee (protocol code 395/2020). All participants gave their written informed consent before inclusion in the study.

### 2.3. Anthropometric Characteristics, Body Composition, and Physical Fitness Assessments

Height was measured by using a stadiometer to the nearest 0.1 cm. Weight, muscle mass (MM), fat mass (FM) body mass index (BMI), and basal metabolic rate (BMR) were measured by hand-to-foot bioelectrical impedance instrument in upright position (InBody R20, Seoul, Korea): for each measurement, subjects’ age, gender, and height were settled on the hand-to-foot bioelectrical monitor. Subjects’ physical fitness (PF) was evaluated using the following tests: handgrip strength test (HG), standing long-jump test (SLJ), YMCA 3-minute bench step test (ST), one-legged stance balance test (OLSB), and reaction time test (RT).

Handgrip test (HG) [24]: Handgrip strength was measured using a Jamar hydraulic hand dynamometer to evaluate muscle strength. Subject was seated on a chair without armrests and held the dynamometer in the hand to be tested, with the arm at right angles and the elbow by the side of the body without touching it. The subject should be strongly encouraged to give maximum effort. The measurement was repeated three times on the dominant hand, with a recovery of 30 seconds from the first measurement to the next one. The average of the three measurements was considered.

Standing long-jump test (SLJ): This test was performed to measure the lower extremity power. The subject stands behind a line marked on the ground with feet slightly apart. A two-foot take-off and landing were used, with bending of the knees to provide forward drive. The subject attempted three times to jump as far as possible, landing on both feet without falling backwards maintaining the arms on the hips. The best of three attempts was considered. 

YMCA 3-minute bench step test (ST): This test was administered according to the YMCA step test procedure (12-inch bench height, step frequency at 96 beats/min). The stepping frequency was indicated by the metronome, and the trial lasted for three minutes. During and three minutes after the test, heart rate was continuously measured using a chest belt device (RS400, POLAR Electro, Germany). After, test subjects were seated. The one-minute heartbeat count (1 min-HBC) as defined by the original YMCA step test was approximated, calculating the mean of twelve consecutive POLAR heart rate records in 5 s intervals, starting 5 s after workload termination. VO_2max_ (ml/kg/min) was then calculated as previously reported [25].

One-legged stance balance test (OLSB) [26]: Subjects, without shoes, had to stand unassisted on one leg with closed eyes and were recorded in seconds from the time one foot was flexed off the floor to the time when it touched the ground or the standing leg or an arm left the hips. Two measurements were taken for each limb, and the best attempt was recorded.

Reaction time test (RT): Reaction time was assessed as previously reported by Eckner et al. [27]. The subject was seated with the arm resting on a table in a comfortable position, and they then caught the apparatus as quickly as possible after it began to fall. The fall distance was measured and then converted into a reaction time (in milliseconds) using the formula for a body falling under the influence of gravity (d 5 ½gt^2^), where *d* is distance, *g* is acceleration due to gravity, and *t* is time.

### 2.4. Statistical Analysis

The sample size of 24 was used for the statistical power analyses. The effect sizes and the alpha level used for this analysis were 0.3 and 0.05, respectively. The post hoc analyses revealed that statistical power for this study was 0.8 for detecting a medium effect (G*power 3.1). All descriptive data are reported as mean ± SD. Correlation analysis was used to explore the relationships between RT, body composition, and physical fitness variables. A repeated measures ANOVA (RM-ANOVA) was used for RT and PF variables with time as within-participants factor (T_0_ and T_6_) and gender as between-participant factors (males vs. females). Seeing that RT was significantly different after dance practice (T_6_), a RM-ANOVA was used for RT with time as the within-participants factor (T_0_, T_6,_ and T_10_) and gender as the between-participant factor (males vs. females). Post hoc analysis with Bonferroni correction was performed to assess differences in RT between T_0_, T_6_, and T_10_. Statistical analyses were conducted using SPSS v. 23 (IBM International, Chicago, IL, USA), and the level of significance was established at *p* ≤ 0.05.

## 3. Results

**Figure 1:**
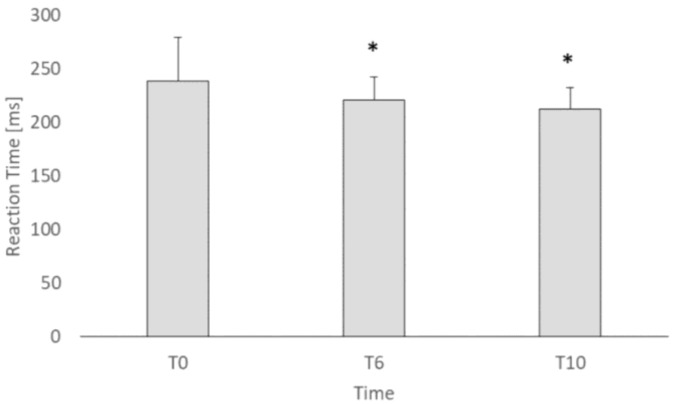
Subjects’ reaction time (ms) pre (T_0_) and post (T_6_) dance intervention and after summer season (T_10_). * *p* < 0.05 vs T_0_.

The drop-out rate was 22.6% at T_6_ and 42% at T_10_. The significant main effect of time was found for RT (F_1,22_=16.8, *p* < 0.01, ηp^2^0.43). In detail, RT at T_6_ was 9% faster than T_0_. No gender differences were found between males and females in the RT parameter. (Table 1). No significant time x gender interaction was found for all the variables. Moreover, significant gender differences were found in weight (*p* = 0.003), muscle mass (*p* < 0.01), percent of fat mass (*p* < 0.01), and hand grip (*p* < 0.01) (Table 2). Indeed, females showed lower weight, muscle mass, and hand grip values, while a higher percentage of fat mass compared to males (Table 2). Moreover, RT was significantly correlated to VO_2max_ and OLSB at T_0_ and to VO_2max_, OLSB, and SLJ at T_6_ as reported in Table 3. Seeing that RT was significantly different between T_0_ and T_6_, this variable was the only one re-evaluated after four months (T_10_) in 18 subjects. A significant effect of time was found for RT (F_2,16_ = 6.59). Post hoc analysis showed that RT was significantly higher at T_0_ (239.1 ± 40,7 ms) than T_6_ (221.2 ± 20.3 ms) and T_10_ (212.0 ± 21.9 ms) as shown in Figure 1.

## 4. Discussion

The aim of the present study was to investigate the effects of six months of ballroom dance on physical fitness (PF) and reaction time (RT) in middle-aged dancers. Results showed that dance training had a significant effect on RT, while no differences were found for the other dependent variables. Specifically, RT values were statistically lower at T_6_ and T_10_ than T_0_.

Results showed gender differences regarding anthropometric measures. In particular, females had lower weight, muscle mass, HG, and higher percent of fat mass than males. Flanagan and colleagues [28] highlighted that sex-specific differences in PF are already noticeable before pubescence. Regarding HG values, subjects showed higher values than those reported by Emerenziani et al. [29] and Vaccaro et al. [16]. This difference could depend on the younger age of the subjects involved in the present study compared to those involved in Vaccaro et al. [16]. Indeed, the latter study [16] showed that experienced older adults had a value of HG equal to 23.2 kg_f_ at pre and 23.8 kg_f_ after dance intervention. 

Regarding cardiorespiratory fitness, VO_2max_ values of enrolled male and female dancers were good and excellent according to the ACSM Health-Related Physical Fitness Assessment Manual [30]. These values were higher than those reported by Kattenstroth et al. [20] and by Huang et al. [31]. These differences could be justified by the different age and different expertise between the studies considered. Indeed, Fleg et al. [32] showed that maximum oxygen consumption has an accelerated rate of decline after the age of 60, while our dancers mean age was 59.1. In addition, in the study by Kattenstroth et al. [20], the non-significant effect of dance practice on cardio-respiratory functions might be justified by the limited amount of weekly training of the intervention (1h/wk.) Although, in the present study, the amount of training was 4.5 h/w, no significant improvements on PF were found as well. We might hypothesize that our experienced dancers had previously reached their PF plateau due to their multi-year practice dancing activities. Thus, to elicit further improvements, a greater exercise intensity and volume than that proposed should be necessary. 

OLSB results indicate no differences after the intervention in contrast with Rehfeld et al. [33] and Sohn et al. [34], who reported improved balance and sensorimotor abilities and improved static and dynamic balance in healthy and active older adults. As previously suggested [16], this difference may account on the higher technical ability of our dancers compared to the beginners and/or unhealthy ones. 

RT showed a significant and negative correlation with VO_2max_ and OLSB at T_0_ and with VO_2max_, OLSB, and SLJ at T_6_. Therefore, we could hypothesize that better cardiorespiratory fitness, balance, and lower limbs muscle power lead to a better RT result. Results are in agreement with those reported by Ando S et al. [35] showing that the increase in the RT is negatively correlated with maximal oxygen uptake VO_2max_. Moreover, it has been showed that balance training improves RT in healthy older adults [36], highlighting the positive correlation between balance and RT. Last, as previously reported [37], muscle power might influence RT positively. However, in the present study, this correlation was found only at T_6_. Further studies with a higher number of participants will deeply investigate these correlations.

Regarding the RT, a significant improvement was found after dance intervention. Indeed, the average RT was 239 at T_0_ and 217 ms at T_6_, suggesting that experienced dancers also present faster RTs at baseline than inexperienced dancers due to multi-year dance practice. These results are in agreement with those reported by Kattenstroth and colleagues [20] who found an improvement in RT after non-partnered dance in older dancers. However, Kattenstroth et al. [20] did not evaluate whether the positive effects of dance practice on RT would also be maintained after a period of unstructured activity. Conversely, since RT was the only variable that improved after dance intervention, we re-evaluated RT 4 months after the end of dancing practice (summer season) (T_10_) to verify whether this improvement had been maintained. Faster RT was maintained at T_10_ (as shown in Figure 1), suggesting that the unstructured dance practice during summer season might have maintained the positive effects of dance. Teixeira-Machado et al. [21] suggested that dance can improve functional brain plasticity, integrating different brain areas that induce both structural and functional changes. In addition, Hänggi et al. [38] proposed that anatomical differences between dancers and non-dancers are a consequence of the relative duration and intensity experience of professional dancing. Thus, it could be hypothesized that our dancers have maintained faster RT after a 4-month period of break because of their ability to maintain dance practice adaptations. In this regard, it would be interesting to evaluate specific brain areas in future studies to monitor long-lasting ability to retain positive neural adaptations even with a low-impact activity after a break from practice. 

Moreover, according to Müller al. [22], a long-term dancing intervention (18 months) in healthy elderly individuals could be better than tedious physical exercise in inducing neuroplasticity in the aging brain, due to the multimodal idea of moving. In addition, the simultaneous training of cognitive and physical abilities, which is proper for dancing, may offer greater benefits on daily life functioning.

The authors are aware of some study limitations. First of all, the number of subjects involved in this intervention study could be extended to a wider population with the presence of a control group. Additionally, adherence to dance classes was not collected as dancers were all experienced showing high levels of participation. However, the significant effect on RT observed in our population, in both T_6_ and T_10_ compared to T_0_, reinforces the strength of the study. It would be of interest to monitor the subjects’ PF for a longer period of dance practice, such as two years, to better evaluate the duration of the effects of ballroom dance on RT. Finally, functional magnetic resonance imaging (fMRI) on the primary (M_1_) and secondary (premotor and supplementary motor areas) cortex could provide useful information on functional changes underlying RT improvements after a long period of dance practice.

## 5. Conclusions

A six-month ballroom dance practice had positive effects on reaction time but no effects on subjects’ PF in experienced middle-aged adults. Moreover, the improvement in RT was maintained four months later. Thus, dance practice could represent an effective strategy for a successful aging. Further studies are needed to investigate different types of dances on PF outcomes and on RT.

## Figures and Tables

**Table 1:** Subjects’ body composition and physical fitness variables pre (T_0_) and after (T_6_) dance intervention.

Variables	T0			T6		
Weight (kg)	71.1	±	13.3	71.5	±	13.7
MM (kg)	26.6	±	6.0	27.1	±	6.3
%FM (%)	32.4	±	6.8	31.4	±	6.4
BMI (kg/m^2^)	26.3	±	4.0	26.4	±	4.0
VO_2max_ (ml/kg/min)	37.0	±	4.8	36.8	±	5.1
HG (Kg_f_)	33.8	±	9.6	33.6	±	9.5
OLSB (s)	4.2	±	3.0	4.9	±	2.9
SLJ (cm)	68.4	±	22.7	69.6	±	21.5
RT (ms)	238.7	±	43.1	217.3	±	27.9 *

MM = muscle mass; %FM = percentage of fat mass; BMI = body mass index; VO_2max_ = maximum oxygen consumption; HG = handgrip test; OLSB = one-legged stance balance test; SLJ = standing long-jump test; RT = reaction time test. * *p* <0.05 vs T_0_.

**Table 2:** Gender differences in body composition and physical fitness variables.

Variables	Males			Females		
Weight (kg)	78.3	±	3.1	63.0	±	3.4 *
MM (kg)	31.2	±	1.0	21.7	±	1.1 *
%FM (%)	28.4	±	1.4	36.0	±	1.6 *
BMI (kg/m^2^)	27.4	±	1.1	25.1	±	1.2
VO_2max_ (ml/kg/min)	37.9	±	1.3	35.7	±	1.4
HG (kg_f_)	40.3	±	1.7	25.9	±	1.8 *
OLSB (s)	4.9	±	0.8	4.1	±	0.8
SLJ (cm)	74.3	±	5.7	62.7	±	6.1
RT (ms)	220.8	±	9.2	236.5	±	10.0

MM = muscle mass; %FM = percentage of fat mass; BMI = body mass index; VO_2max_ = maximum oxygen consumption; HG = handgrip test; OLSB = one-legged stance balance test; SLJ = standing long-jump test; RT = reaction time test. * *p* < 0.05 vs males.

**Table 3:** Correlation between reaction time (RT) and physical fitness variables at T_0_ and T_6_

		Weight (kg)	MM (kg)	%FM (%)	VO_2max_ (ml/kg/min)	HG (kg_f_)	OLSB (s)	SLJ (cm)
RT (T_0_)	r	−0.053	−0.140	0.086	−0.417 *	−0.014	−0.415 *	−0.381
	p	0.806	0.516	0.690	0.042	0.949	0.043	0.066
RT (T_6_)	r	0.012	−0.103	0.191	−0.454 *	0.030	−0.587 * *	−0.698 * *
	p	0.957	0.631	0.370	0.026	0.888	<0.01	<0.01

MM = muscle mass; %FM = percentage of fat mass; VO_2max_ = maximum oxygen consumption; OLSB = one-legged stance balance test; SLJ = standing long-jump test; RT = reaction time test. * *p* < 0.05 and ** *p* < 0.01; r = Pearson’s correlation.

## Data Availability

Dataset will be made available upon request.

## Supplementary Materials

The following are available online at https://www.mdpi.com/1660-4601/18/4/2036/s1, File S1: list of songs for one dance session.
