# Supplementary material for: Effects of Ballroom Dance on Physical Fitness and Reaction Time in Experienced Middle-Aged Adults of Both Genders
Source: Int J Environ Res Public Health. 2021 Feb 19;18(4):2036. doi: 10.3390/ijerph18042036 (PMC7922230; doi:10.3390/ijerph18042036)
Supplement: Supplementary file 1 [file ijerph-18-02036-s001.pdf]

**Waltz: 29 bpm**

- Il carrozzone - Renato Zero
- Still me - Erkan Aki
- The shore of the swilly - Phil Coulter

**Tango: 32 bpm**

- Aj se baila el Tango- Veronica Verdier
- What is love - Haddaway
- The final countdown - Europe

**Viennese Waltz: 59 bpm**

- Nuvole - Patty Pravo
- Serenade - Schubert
- Gramofon waltz - Eugen Doga

**Slow Foxtrot: 29 bpm**

- Back where I belonge
- Dancing Queen - ABBA
- You will never know - Imany

**Quickstep: 52 bpm**

- Bambola - Patty Pravo
- Tu mi porti su - Lorenzo Jovanotti
- Tu Vò fa l'Americano - Renato Carosone
